# Dupilumab opens a therapeutic window in food protein–induced enterocolitis syndrome by unlicensing dendritic cells

**DOI:** 10.1016/j.jacig.2025.100592

**Published:** 2025-10-30

**Authors:** Matthew Plassmeyer, Naomi Enav, Michael Girgis, Mikell Paige, Linda Todd, Laureana Israni, Oral Alpan

**Affiliations:** a Amerimmune, Mclean, Va; b Department of Bioengineering, George Mason University, Fairfax, Va; c Department of Chemistry, George Mason University, Fairfax, Va

**Keywords:** Food protein–induced enterocolitis syndrome, dendritic cell, OX40L, T_H_2, dupilumab, CRTH2

## Abstract

**Background:**

Food-protein–induced enterocolitis syndrome (FPIES) is a non–IgE-mediated reaction to food antigens for which no disease-modifying treatment exists. The mechanistic heterogeneity of FPIES—spanning multiple incompletely understood immune pathways—has hindered the prioritization of therapies in clinical trials.

**Objective:**

In this observational study, we sought to determine whether dupilumab, an mAB that is an IL-4 receptor-alpha antagonist, can induce clinical remission in FPIES.

**Methods:**

We describe (1) a detailed single-patient case of wheat-triggered, endoscopy-confirmed colitic FPIES treated with dupilumab 300 mg subcutaneously every 2 weeks and (2) a prospective follow-up of 7 additional patients with FPIES all of whom initiated dupilumab for FDA approved comorbidities as a part of their clinical care. Serial flow cytometry quantified dendritic-cell OX40-ligand (OX40L) and CD8^+^ chemoattractant receptor-homologous molecule positive T-cell subsets before and after treatment; open food challenges assessed clinical tolerance.

**Results:**

*Index case*: Within 2 injections of dupilumab, the wheat-sensitive patient tolerated a 50-g wheat protein challenge without gastrointestinal symptoms—first uneventful exposure in 20 years. Discontinuation of dupilumab led to relapse; reinitiation again restored clinical tolerance. OX40L^+^ dendritic cells (DCs) fell from 22% to 8% to 10% and remained stable, whereas circulating chemoattractant receptor-homologous molecule positive CD8 (T cytotoxic 2) cells increased to approximately 12%, suggesting redistribution of these T cells from the gut tissue. *Cohort:* All 7 additional patients (ages 2-58 years; triggers: milk, soy, rice, wheat, shellfish) achieved unrestricted dietary tolerance within 3 months, mirroring the immunophenotypic trajectory of the index case. All patients had provided consent under Western Institutional Review Board (protocol no. 20121950).

**Conclusions:**

Dupilumab rapidly and durably abrogated food reactivity across diverse FPIES phenotypes associated with downregulated dendritic-cell OX40L. These findings identify the IL-4/IL-13–OX40L axis as a tractable therapeutic target and support formal trials of dupilumab—including high-dose “rescue” regimens—in acute and chronic FPIES.

## Introduction

Food-protein–induced enterocolitis syndrome (FPIES) is a food-induced inflammatory disease of the gastrointestinal tract in the absence of IgE sensitization.^1^ Classically, it presents in infants as an abrupt, self-limited emetic crisis 1 to 4 hours after ingesting staples such as cow’s milk, soy, rice, or oats. In some cases, the episodes can be severe with pallor, lethargy, and repetitive projectile vomiting (with or without loose stools), but resolve completely between attacks, and so caregivers often mistake them for “acute food poisoning.”^2^ Diarrhea is infrequent, and most children remit spontaneously by age 3 to 5 years.^3^ Accordingly, the 2017 international consensus criteria focus on this vomiting-dominant phenotype for diagnosis and research harmonization.^1^

Adults diverge sharply, with a female predominance, absent vomiting in approximately 25% of cases, and a shift in trigger foods from mammalian milks/grains to crustaceans and bivalves.^4^ Moreover, adult FPIES often shows a chronic pattern: after exposure, severe crampy pain, watery or bloody diarrhea, and radiologic or endoscopic colitis, persisting up to several days.^5^ Diagnosis therefore demands vigilance. Key features are reproducible delayed (1-6 hours) gastrointestinal symptoms—dominant cramping and diarrhea, occasional emesis—after ingestion of a single identifiable food, rapid improvement with avoidance, and negative food-specific IgE. Because the 2017 criteria mandate vomiting, strict adherence risks underdiagnosis; meticulous dietary history and, when safe, supervised oral food challenge remain the criterion standard and may obviate colonoscopy. Laboratory findings are nonspecific transient neutrophilia or thrombocytosis; no serum biomarker yet distinguishes FPIES from other conditions that cause postprandial colitis.^1^^,^^2^^,^^4^ Awareness that an allergic process can masquerade as inflammatory bowel disease prevents misdiagnosis, invasive workups, and unwarranted immunosuppression.

Mechanistic studies show findings in both the innate and adaptive immune response. The innate immune arm shows brisk activation of neutrophils, eosinophils, monocytes, and natural killer and dendritic cells with surges of TNF-α, IL-6, IL-8, and CXCL8 on food allergen exposure.^6^ On the adaptive side, circulating T cells upregulate CD69, accumulate in lamina propria, and express a T_H_17 and/or T_H_2 (IL-4, IL-5, IL-13) phenotype.^7^ More than 50% of patients harbor eczema, asthma, or rhinitis, supporting this T_H_2 bias.^8^ Interrupting either the adaptive or innate arm is attractive, therapeutically.

Dupilumab is a fully human anti–IL-4 receptor-alpha (IL-4Rα) mAb that blocks IL-4/IL-13 signaling and is approved for multiple atopic disease indications.^9^ A recent study of dupilumab in IgE-mediated food allergy has failed.^10^ However, B-cell class switch to IgE is not the only function of IL-4.^11^ For this reason, we decided to explore the role of dupilumab in FPIES. We report a middle-aged man with wheat-triggered adult-onset colitic FPIES in whom dupilumab produced rapid immunologic changes and durable clinical tolerance, providing proof-of-concept for successful IL-4Rα antagonism in non–IgE-mediated food allergy.

## Results and discussion

A 52-year-old French-born chiropractor, resident in the United States since age 21 years, was referred for refractory eczema and wheat-associated lower gastrointestinal symptoms labeled “ulcerative colitis” 20 years earlier. At age 28 years, he began experiencing abrupt crampy pain followed 1 to 3 hours later by profuse watery diarrhea (4-8 large-volume stools over 12 hours) with visible blood and mucus. Wheat-containing foods (bread, pasta, pastries) invariably precipitated attacks; there was never urticaria, angioedema, wheeze, pruritus, or oral tingling. Between episodes he was well.

At age 30 years, skin prick testing result and serum specific IgE panels were negative. Two years later, a severe flare required emergency hydration. Labs showed normal hemoglobin, mild neutrophilia, elevated C-reactive protein/erythrocyte sedimentation rate, and normal total IgE. Colonoscopy revealed mild patchy erythema in sigmoid and descending colon; histology showed focal cryptitis, mild architectural distortion, and modest lamina-propria eosinophils (<20 hpf) without basal plasmacytosis, granulomas, or dysplasia—consistent with food-driven injury. Mesalamine was prescribed empirically, but remission followed only strict wheat elimination; upper endoscopy was normal.

Over the next decade, gluten avoidance controlled symptoms. Four accidental open challenges (soy sauce, craft beer, beignets, home-cooked pasta) reproduced identical attacks within 4 hours, whereas minor cross-contact (shared toaster) was tolerated, indicating a threshold effect rather than extreme sensitization.

At age 50 years, he developed pruritic, lichenified plaques on his torso, antecubital areas, face, and neck unresponsive to high-potency topical steroids (SCORing Atopic Dermatitis 30). Baseline height 178 cm, weight 79 kg (body mass index, 25 kg/m^2^), blood pressure 110/78 mm Hg, pulse 68, temperature 36.6°C; abdominal exam benign. Labs (Table I) showed normal complete blood cell count, metabolic panel, serum IgE, and tissue-transglutaminase IgA. Skin prick test results for food/environmental allergens were negative. There was no eosinophilia. Lymphocyte phenotyping revealed normal T/B/normal killer cell counts but increased CD8-cell caspase-1 expression and CD8 effector-memory T cells. There was no family history of celiac disease, inflammatory bowel disease, or eosinophilic gastrointestinal disease, although a half-sister reported intermittent mild hematochezia.

**Table I:** Patient characteristics

Test (units)	Result	Reference range
Complete blood cell count		
White cell count (×10^3^/μL)	4.9	3.4-10.8
Hemoglobin (g/dL)	13.7	13.0-17.7
Platelets (×10^3^/μL)	262	150-450
Absolute neutrophils (×10^3^/μL)	2.9	1.4-7.0
Absolute lymphocytes (×10^3^/μL)	1.4	0.7-3.1
Basic metabolic panel		
Creatinine (mg/dL)	1.04	0.76-1.27
BUN (mg/dL)	15	6-24
Calcium (mg/dL)	9.6	8.7-10.2
AST/ALT (IU/L)	16/15	0-40/0-44
Inflammatory markers		
C-reactive protein (mg/L)	4	0-10
Erythrocyte sedimentation rate (mm/h)	8	0-30
Autoantibodies		
ANA titer, pattern	1:40, speckled	<1:40
Immunoglobulins		
IgG (mg/dL)	1108	603-1613
IgA (mg/dL)	269	90-386
IgM (mg/dL)	132	20-172
Total IgE (IU/mL)	12	6-495
Allergen-specific IgE		
Wheat IgE (kU/L)	<0.10	<0.10
Complement		
C3 (mg/dL)	116	82-167
C4 (mg/dL)	29	14-44
Flow-cytometric immunophenotyping (selected abnormalities)		
Activated caspase-1 in CD8^+^ cells (%)	39.5	<10
CD8^+^ Tem (CD45RO^+^CCR7^-^) (%)	73.2	11.0-53.7

*ALT*, Alanine transaminase; *ANA*, antinuclear antibody; *AST*, aspartate transaminase; *BUN*, blood urea nitrogen.

Dupilumab 300 mg subcutaneously every 2 weeks was initiated for atopic dermatitis. After 2 injections he consumed a wheat baguette in France and, for the first time in a decade, experienced no gastrointestinal reaction. Encouraged, we conducted 3 further open challenges (3-4 days escalating wheat ingestion, cumulative 15-50 g protein via croissants, bread, pancakes, baguette); all were uneventful.

Six months later, insurance interruptions forced discontinuation. Within 8 weeks he experienced a classic flare after homemade pizza abroad—crampy pain, bloody diarrhea—resolved by a 2-week prednisone taper (40 mg daily then taper). Once coverage resumed, dupilumab was restarted; 1 day after the first dose, he underwent a supervised wheat challenge with commercial pasta and remained symptom-free for 48 hours, confirming persistence of wheat reactivity absent treatment. Patient continues consuming wheat with no symptoms on dupilumab.

**Fig 1:**
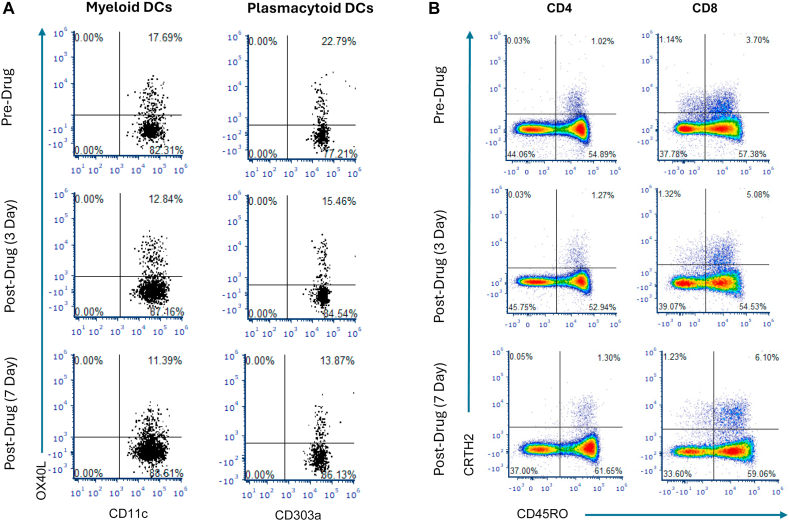
DC OX40L and CRTH2^+^ T-cell shifts with dupilumab. Whole blood collected in heparin tubes was immune stained per the clinical standard immunophenotyping protocol (Amerimmune LLC, Fairfax, Va). In brief, for DC staining, 200 μL of whole blood was stained with the following antibody combinations (lineage consisting of CD3, CD14, CD16, CD19, CD20, CD56, and CD34-FITC): CD11C-PE, HLA-DR-PerCP eF710, CD303a-APC, and OX40L-PECy7 for 30 minutes at 4˚C. To stain CRTH2 cells, 100 μL of whole blood was stained with the following antibody combination: CD4-FITC, CD45RO-PerCPCy5.5, CRTH2-APC, CD3-AF700, CD8-SB436, and CD45-EF506 for 30 minutes at 4˚C. Cells were acquired on an ATTUNE NXT (ThermoFisher Scientific, Waltham, Mass). **A,** DCs are identified as HLA-DR^+^Lineage^−^CD34^−^ cells. DCs are separated into mDCs and pDCs as CD11c^+^CD303a^−^ and CD11c-CD303a^+^, respectively. mDC and pDC OX40L^+^ cells were identified on OX40L/CD11c and OX40L/CD303a plots. **B,** T cells were identified as CD45^+^CD3^+^ cells. The CD4^+^CRTH2 and CD8^+^CRTH2 populations were subsequently identified using CRTH2 vs CD45RO plots gated on CD4^+^ cells and CD8^+^ cells, respectively. *APC*, Antigen-presenting cell; *FITC*, fluorescein isothiocyanate; *PE*, phycoerythrin.

**Fig 2:**
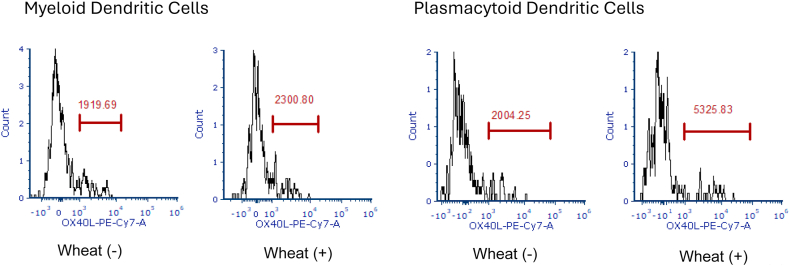
Wheat-induced OX40L expression on DCs. PBMCs are incubated with PBS or wheat for 2 hours. Cells are then stained to identify myeloid (CD11c) and plasmacytoid (CD303a) DCs. OX40L expression is shown on the x-axis. Incubation with wheat (100 ng/mL) increases the mean fluorescence of OX40L, an indication of increased number of OX40L molecules on the surface of cells. *PE*, Phycoerythrin.

Serial flow cytometry at dupilumab reinitiation showed dendritic-cell (DC) OX40-ligand (OX40L) falling from 22% to 12% within 1 week. Meanwhile chemoattractant receptor-homologous molecule positive (CRTH2^+^) CD8 (T cytotoxic 2 [Tc2]) cells rose from 4% to approximately 10%, with no change in CRTH2^+^ CD4 T_H_2 cells. By 8 weeks, OX40L and Tc2 stabilized at 8% to 10% and approximately 12%, respectively (Fig 1). Pretherapy whole-blood stimulation with wheat extract induced 1.2-fold OX40L upregulation in myeloid dendritic cells (DCs) and 2.6-fold in plasmacytoid DCs, confirming allergen responsiveness, a response that is absent in healthy controls (Fig 2).

**Fig 3:**
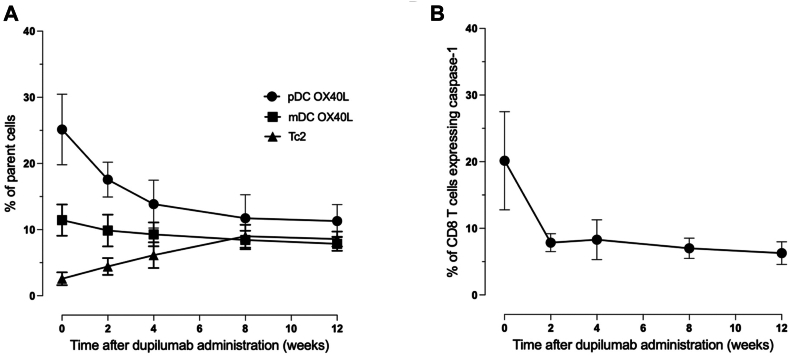
Dupilumab downregulates DC OX40L and CD8 caspase-1 with Tc2 expansion. **A,** Experiments were performed, and cell subsets were gated as described in Fig 1. Percentages for each population—Tc2 cells (CRTH2^+^ CD8^+^ T cells), OX40L-positive plasmacytoid DCs, and OX40L-positive myeloid DCs—were calculated relative to their respective parent populations. CRTH2 is a very sensitive marker for IL-4–secreting T cells.^12^ The decrease in pOX40L (*P* < .0001) and increase in Tc2 cells (.01) are statistically significant (1-way ANOVA). The decrease in mDC OX40L was not as pronounced as in pDCs but still met statistical significance (*P* < .02). **B,** Active **c**aspase-1 staining was performed as described elsewhere.^13^ The reduction is rapid and persistent (*P* < .0001). *mDC*, Myeloid DC; *pDC*, plasmacytoid DC.

Building on our observations from the case report, we then followed 7 additional patients with FPIES, who were started on dupilumab therapy, based on Food and Drug Administration–approved comorbid conditions (Table II). None of the patients had life- threatening FPIES. On clinical follow-up, all patients were able to tolerate all the foods they had significant gastrointestinal reactions to, despite wide variability in food triggers and disease chronicity (6 months to 18 years). The decrease in plasmacytoid DC OX40L along with expansion of circulating CRTH2^+^ Tc2 cells followed the pattern observed in the original case report (Fig 3, *A*).^12^, ^13^ To our surprise, CD8^+^ T-cell active caspase-1 also dropped abruptly (Fig 3, *B*). Even though inflammasome activation through caspase-1 can enhance T_H_2 responses, such effects have not been studied in human disease states.^14^ These data demonstrate the clinical benefit of dupilumab across different clinical forms of FPIES. No serious adverse events occurred in any of the patients. These data underscore both the safety and the broad applicability of IL-4Rα blockade for FPIES remission across a wide age range.

**Table II:** Clinical characteristics and outcome of the additional FPIES cohort

Age; sex	Indication	Foods	Symptom	IgE	Dose	Time to full remission
18 mo; F	Eczema	Milk, soy, egg, all grains	Vomiting in 2 h	2	200 q4w	All foods were reintroduced in a 3-wk period without any symptoms
38 y; M	Prurigo nodularis	Multiple (>10)	Diarrhea and bloating lasting 2-3 d	10	300 q2w	All foods reintroduced in 2-mo period. Patient stated gaining so much weight that he had to start Ozempic
4 y; M	Eczema	Milk	Vomiting	5	300 q2w	Milk reintroduced in 2 wk
30 y; M	Asthma	Milk, soy, wheat	Diarrhea, bloody stool	18	300 q2w	Foods introduced in the order of milk, soy, and wheat over a course of 4 wk
28 y; F	Nasal polyps	Shellfish, fish	Vomiting, stomach pain	122	300 q2w	Passed multifood challenge in 2 wk after therapy
2 y; M	Eczema	Milk	Vomiting in 1 h	19	200 q4	Passed milk challenge in 4 wk after therapy
68 y; F	Asthma	Walnut, cashew, pistachio	Diarrhea, colitis	10	300 q2	Passed challenge in 3 mo

“Foods” lists the culprit allergens provoking FPIES reactions before treatment. “Symptom” records the predominant acute manifestation at the time of reaction. IgE is the total serum IgE concentration, reported in international units per liter (IU/L). Dose refers to the subcutaneous dupilumab loading/maintenance regimen, expressed as milligrams (mg) followed by dosing interval: q2w or q4w. “Time to full remission” indicates the interval from first dupilumab dose to complete, sustained reintroduction of all offending foods without recurrence of symptoms, as documented by oral food challenges or dietary history. “Decrease in pDC/mDC OX40L (%)” represents the percentage reduction from each patient’s pretreatment baseline in OX40-L surface expression on pDC and mDC subsets after therapy completion.

*F*, Female; *FPIES*, food-protein–induced enterocolitis syndrome; *M*, male; *mDC*, myeloid DC; *pDC*, plasmacytoid DC; *q2w*, every 2 weeks; *q4w*, every 4 weeks.

FPIES is an orphan disease with a prevalence of approximately 0.5% to 0.7% in infants and far less frequent in adults with approximately 1 million patients in the United States.^1^ Current management is dietary avoidance plus supportive care during acute crises. Dupilumab therefore satisfies the rarity-severity-no-therapy triad underlying orphan-drug incentives and represents the first plausible disease-modifying biologic for this condition. The first index case presented here provides evidence that the IL-4Rα antagonist dupilumab can rapidly abrogate clinical reactivity in adult wheat-triggered colitic FPIES, challenging the paradigm of lifelong avoidance and emergency-department preparedness.

An important finding in the index case as well as the follow-up cohort is the dupilumab-induced drop in DC OX40L. OX40L is a TNF-superfamily costimulatory molecule induced on DCs and other antigen-presenting cells. When DC-expressed OX40L engages OX40 on recently activated CD4^+^ and CD8^+^ T cells during antigen presentation, it augments TCR/CD28 signaling to enhance NF-κB and PI3K–protein kinase b pathways, driving expansion, survival, cytokine production, and the stabilization of memory.^15^ In naive CD4^+^ cells, OX40L potently skews differentiation toward type-2 programs (eg, GATA binding protein-3, IL-4, and IL-13). Epithelial alarmins and licensing cues that are relevant to food reactions—thymic stromal lymphopoietin, IL-33, IL-25—as well as CD40 ligation and microbial/Toll-like receptor signals, upregulate OX40L on mucosal DCs.^16^ In FPIES, where antigen exposure occurs across a stressed but non-IgE milieu, we propose that OX40Lhi gut DCs sustain type-2 cytokine and chemokine production, and reinforce CRTH2-tuned cell trafficking.

**Fig 4:**
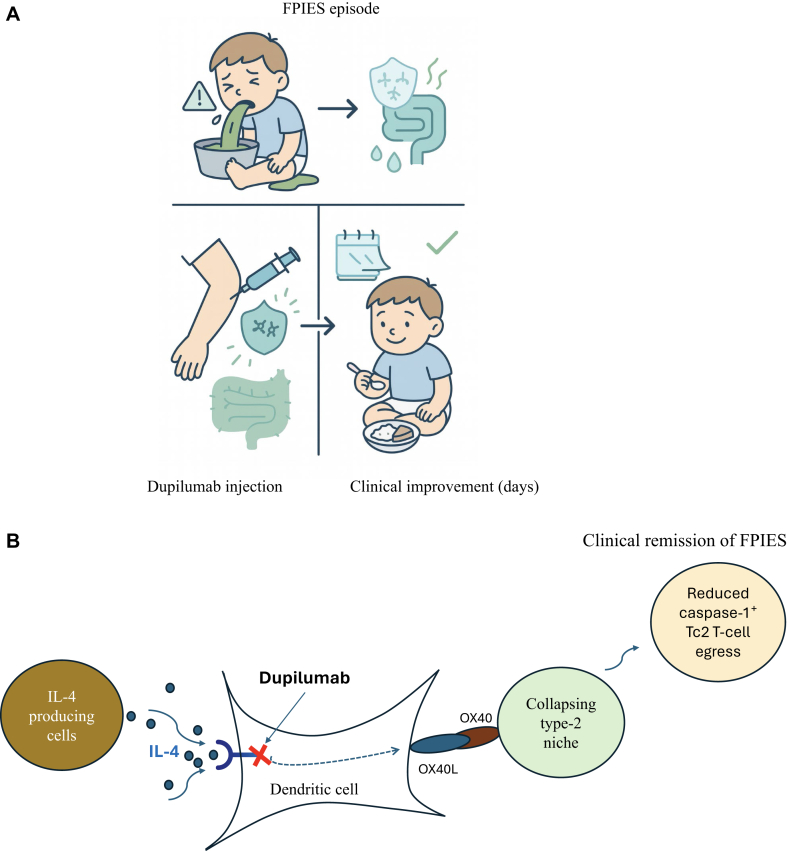
Proposed model for dupilumab’s effect in FPIES. DCs act as a bridge to delicense FPIES-causing T cells. Dupilumab reduces IL-4Rα–STAT6 signaling and DC OX40L, curtailing *new* T_H_2/Tc2 priming; the transient rise in circulating CRTH2^+^ Tc2 reflects egress from a collapsing type-2 niche and serves as a pharmacodynamic indicator of on-target pathway shutdown.

We hypothesize that by blocking IL-4Rα, dupilumab shuts down IL-4–driven DC “licensing.” The sustained decline in OX40L indicates an adaptive-to-innate feedback that retunes antigen-presenting cell costimulation at the DC–T-cell synapse, consistent with Matzinger’s model.^17^^,^^18^ This reset can potentially help extinguish the T-cell program that drives FPIES pathology. Precedent for this directionality exists but is uncommon: for example, CTLA4-Ig, though designed to block T-cell costimulation, delivers reverse signals through CD80/86 on DCs, inducing tolerogenic programs (eg, indoleamine-2,3-dioxygenase) and dampening IL-12/23; likewise, B-cell depletion with rituximab reduces immune-complex stimulation of plasmacytoid DCs, collapsing the type-I interferon cascade.^19^^,^^20^ Our data place dupilumab’s effect in this select class, showing that precisely targeting the adaptive arm can predictably reprogram innate checkpoints via DCs (Fig 4).

Increase in peripheral blood Tc2 cells has been described in asthmatic patients receiving mepolizumab.^21^ These circulating Tc2 cells shift toward a central-memory phenotype (CD45RA^-^ CD62L^+^). In the small dupilumab cohort within the same study, Tc2 cells did not increase; instead ILC2 increased. In skin, dupilumab clears inflammation but disease-linked Tc2 (and T_H_2A) populations persist during clinical remission, consistent with tissue-resident memory rather than active effectors. Even though we did not measure whether the increased Tc2 cells in our FPIES cohort represent effector- or central-memory cells, the decline in caspase-1 more likely reflects reduced effector activity, in line with the mepolizumab data.

Previous studies of dupilumab pharmacokinetics show near-complete IL-4Rα occupancy within hours.^22^ Flow cytometry in moderate to severe AD revealed complete loss of surface IL-4Rα and abolished IL-4–induced STAT-6 phosphorylation as early as 2 hours after 600 mg subcutaneous loading—>95% occupancy.^23^ Furthermore, administration of dupilumab during active FPIES colitis may blunt ongoing IL-4/IL-13–driven OX40L upregulation and resolve the acute disease. Prospective trials embedding a high-dose dupilumab “rescue” arm in emergency FPIES protocols are needed to refine timing, dosing, and safety.

Clinically, the ability to introduce dupilumab early in the course of FPIES—immediately after the first reaction or during a high-risk reexposure period—has 3 tangible advantages: (1) it interrupts fresh IL-4/IL-13–dependent priming in the acute stage, lowering the risk and severity of subsequent delayed colitis-type reactions and reducing emergency room visits, intravenous fluids, and steroid use; (2) it provides a preventive “cover” while families reestablish nutrition, enabling supervised reintroductions sooner and with greater safety; and (3) it may create a therapeutic window for true tolerance acquisition rather than indefinite avoidance by collapsing the type-2 licensing milieu. The clinical cases confirm effective dosage, but also the possibility of PRN (as needed) use in adults to prevent or treat FPIES episodes.

A subset of food-triggered, IgE-negative irritable bowel syndrome can show reproducible meal-provoked symptoms with innate/type-2 immune activation (eg, mast-cell/eosinophil involvement) rather than classic IgE mechanisms.^24^^,^^25^ This phenotype is mechanistically adjacent to FPIES—both likely depend on non-IgE antigen presentation and type-2 licensing cues (eg, DC OX40L). Future studies should investigate shared mechanisms between food-induced irritable bowel syndrome and FPIES as well as explore targeting OX40L, OX40, and IL-4Rα therapeutically for these conditions.

Finally, a prospective proof-of-concept study of dupilumab in FPIES is needed. If successful, a follow-up phase 3 trial can potentially lead to a much-needed therapeutic for FPIES.

**Key messages**

• **Dupilumab treats FPIES, enabling symptom control and safe food rechallenge.**
• **Rapid DC OX40L drop on dupilumab tracks tolerance, an actionable response biomarker.**
• **Tc2 (CRTH2^+^ CD8^+^) rise with CD8 caspase-1 fall coincides with remission, a practical PD (pharmacodynamic) readout.**

## Disclosure statement

Disclosure of potential conflict of interest: All the authors declare that they have no relevant conflicts of interest.
